# Choledochal cyst as a diagnostic pitfall: a case report

**DOI:** 10.1186/1752-1947-2-5

**Published:** 2008-01-14

**Authors:** Uta Waidner, Doris Henne-Bruns, Klaus Buttenschoen

**Affiliations:** 1 Department of General, Visceral, and Transplantation Surgery, University Hospital Ulm, Germany

## Abstract

**Introduction:**

Choledochal cysts are rare congenital anomalies. Their diagnosis is difficult, particulary in adults.

**Case presentation:**

This case report demonstrates the diagnostic and therapeutic pitfalls.

**Conclusion:**

To prevent cost-intensive and potentially life-threating complications, a choledochal cyst must be considered in the differential diagnosis whenever the rather common diagnosis of a hepatic cyst is considered.

## Introduction

Choledochal cysts are rare congenital, but not familial, anomalies of the intrahepatic or extrahepatic biliary tract. Cystic dilatation may affect every part of the biliary tree and may occur singly or in multiple numbers. The incidence in the population is 1:100000 to 1:150000 [1]. The clinical classification, which describes five different types and subtypes, was revised in 1977 by Todani and colleagues [2]. The most common cystic dilatation is type I with diffuse or segmental fusiform dilatation of the common bile duct. This type accounts for 50 to 85% of cases. Type I cysts should be considered in the differential diagnosis of any patient with ductal dilatation.

The leading symptoms include cholestatic jaundice and abdominal pain. A palpable abdominal mass occurs in less than 20% of the cases. In adults, chronic and intermittent abdominal pain is the most common symptom. Recurrent cholangitis and jaundice may also occur. A choledochal cyst is rarely symptomatic, but should be considered if dilatation of the bile duct or the ampulla is demonstrated.

The main diagnostic tool for detection of a choledochal cyst, especially in childhood, is ultrasonography. In adults, computer tomography can confirm the diagnosis; however, endoscopic retrograde cholangiography or magnetic resonance cholangiography are the most valuable diagnostic methods and can accurately show cystic segments of the biliary tree [3].

Surgery is the treatment of choice for a choledochal cyst. Complete excision of all cystic tissue is recommended because of the risk of recurrent cholangitis and the high risk of malignant degeneration [4]. Excision of the cyst and reconstruction of the biliary tree by choledochal/hepato-jejunostomy with a Roux-en Y-loop is the standard procedure [5].

In comparison, simple congenital hepatic cysts are very common. Their incidence is 1:40 in the population and simple congenital hepatic cysts represent the most important differential diagnosis [6]. These cysts are also rarely symptomatic. They are detected incidentally during an operation or by diagnostic measures for other conditions and generally do not require treatment [5]. If symptoms occur in the case of larger cysts, non-specific upper abdominal discomfort and a palpable abdominal mass are most common [7]. Symptomatic cysts can be treated by non-operative invasive intervention or by an operative procedure. Operative procedures comprise cyst fenestration, partial or total cyst resection, and hepatic resection. Laparoscopic cyst fenestration is the treatment of choice because it is a simple and effective procedure with a low mortality [5].

Our case report of a young female with a choledochal cyst emphasizes the difficulties of arriving at the correct diagnosis and documents the efficacy of surgical treatment.

## Case presentation

A 19-year-old Russian woman (height, 1.69 m; weight, 54 kg) with non-specific upper abdominal pain presented to a local hospital for evaluation. She complained of recurrent pain for weeks. Clinical examination revealed neither jaundice nor a palpable abdominal mass. The clinical laboratory data were normal.

**Figure 1:**
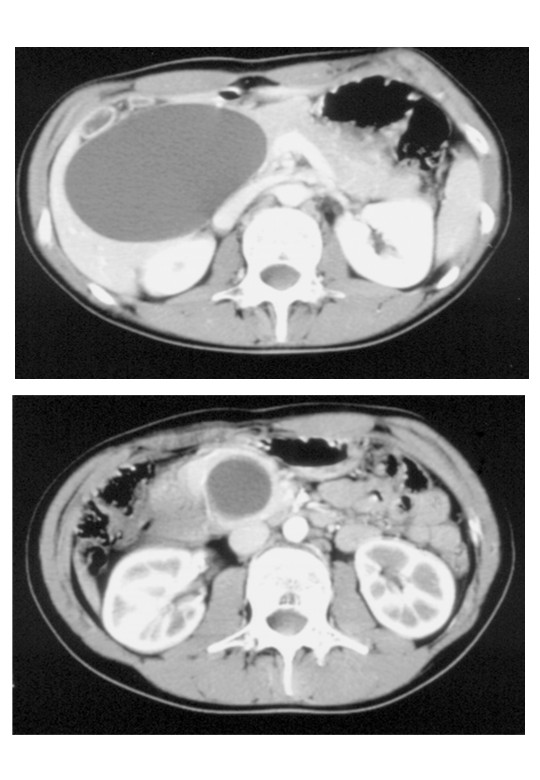
Computer tomography scans: Upper panel: A large cyst,11 cm in diameter, was considered to represent a common hepatic cyst because it was in direct contact with the liver. Lower panel: The dilated distal common bile duct was also misinterpreted as a second hepatic cyst.

Ultrasonography revealed a hypoechogenic, nearly spheric, homogenous formation with a smooth contour in direct contact with the underside of the liver and without any intermediate layer. The finding was most compatible with a large hepatic cyst. Computer tomography showed a clearly limited, hypodense, homogenous structure with a transverse diameter of 11 cm in the immediate vicinity of the liver, anterior to the right kidney, and posterior to the gall bladder (Fig. 1, upper panel). Cystic echinococcosis was excluded serologically. The documented adjacent lower computer tomography-slice depicted a similar hypodense structure, which was nearly circular and only 3 cm in diameter. The larger structure was interpreted as a congenital hepatic cyst due to the direct contact to segment 5 of the liver. The smaller structure was judged as an independent hepatic cyst because it resembled the large cyst, except for its smaller size (Fig. 1, lower panel). Further diagnostic procedures were not performed because the computer tomography was considered sufficient.

Because of the recurrent pain, a laparoscopic fenestration of the large cyst was recommended and this was performed at a primary care hospital. During the procedure, the cyst was approached via the inferior border. The cyst was in direct contact with the underside of segment 5, and the surgeon had no doubt about the liver as the origin of the cyst. A second cyst could not be identified.

A puncture was performed, which resulted in the evacuation of more than 100 ml of bile. Then, the cyst was opened by a 4 × 3 cm incision. Laparoscopic evaluation of the inner cyst revealed two bile ducts and, under the assumption of eroded bile ducts, clips were attached to effect closure. After fenestration, a drain was placed into the abdomen.

The drainage was consistent with a biliary leakage on the second postoperative day. Bilirubin increased to 6.21 mg/dl and the patient developed jaundice. An endoscopic retrograde cholangiography showed a massive dilatation of the distal common bile duct. The injected radiopaque material leaked into the abdomen. The intrahepatic bile system could not be detected. A stent from the duodenum into the dilated bile duct was inserted.

**Figure 2:**
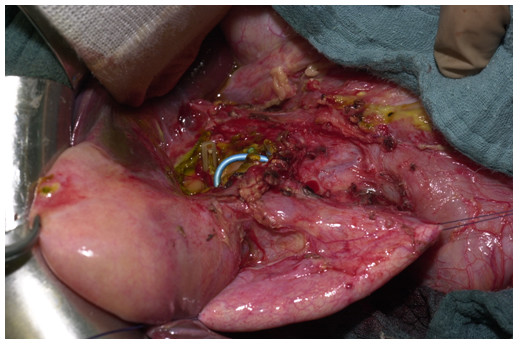
Intraoperative situs: The choledochal cyst was mobilized and fixed with holding sutures. Clips are seen in the cyst, which closed the right and left hepatic ducts.

**Figure 3:**
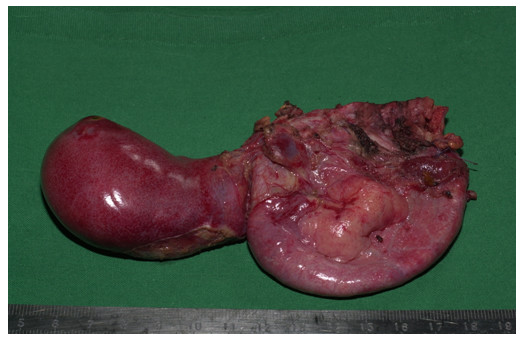
Resected specimen: The gallbladder (left) and the deflated bile duct cyst were removed (right).

Due to these ambiguous findings, the patient was transferred to our university hospital on the third postoperative day. Computer tomography showed incipient pancreatitis. After re-evaluation of the original computer tomography, a large choledochal cyst involving the distal part of the common bile duct was recognized. The patient underwent repeat surgery on the fourth day after the original surgery, and a large choledochal cyst, Todani type 1A, with a diameter of 8–10 cm was found (Fig. 2). The distal end of the stent was palpable in the duodenum, whereas the other end was visible in the fenestrated cyst (Fig. 2). After further exploration of the choledochal cyst, the clips became visible in the cyst (Fig. 2). However, these clips had not closed the suspected fistular ducts, but had occluded the right and left hepatic ducts (Fig. 2). The clips were removed. The cyst was completely excised and the distal common bile duct was closed (Fig. 3). A hepatojejunostomy was performed by a Roux-en-Y loop as the curative therapy.

## Discussion

This case report highlights the difficulties involved in making a correct diagnosis and the operative treatment for a choledochal cyst. Hepatic cysts are much more common and their pathologic and clinical characteristics often overlap with that of choledochal cysts.

Choledochal cysts are rare abnormities of the biliary tree and so may be frequently overlooked in the differential diagnosis.

The non-specific symptoms of choledochal cysts, including pain in the upper abdomen and jaundice, are common in many other illnesses of the upper gastrointestinal tract. The clinical triad of jaundice, a palpable mass and abdominal pain occurs only in one-third of all patients. Abdominal pain is the prominent complaint in adults, which also led our patient to seek medical attention. The choledochal cyst (1:100000) was easily mistaken, as may frequently happen, for a much more common solitary congenital liver cyst (1: 1000), especially if typical symptoms are absent in a large cyst [8,9].

Ultrasonography is usually the first examination and is very sensitive in the detection of cystic structures, but rather non-specific in identifying their origin. The diagnosis of the choledochal cyst in our case report may have been missed because the technical quality of the examination may not have allowed for recognizing the anatomic pathology. A computer tomography usually can give more information and modern techniques, including reconstruction, should allow for establishing the diagnosis. However, the radiologist did not ascertain any signs of separation because the choledochal cyst had immediate contact with the liver and a simple liver cyst was suggested. Endoscopic retrograde cholangiography or magnetic resonance cholangiography can precisely visualize the extrahepatic bile duct and these are the most specific diagnostic procedures. Since endoscopic retrograde cholangiography and magnetic resonance cholangiography are more invasive, they were not done in this case because the computer tomography and ultrasonography findings were considered valid and reliable.

The treatment of a choledochal cyst has changed. In the past, a cysto-jejunostomy was the standard procedure. Currently, excision of the cyst and reconstruction by hepatojejunostomy is the standard therapy [10].

This case report also demonstrates the intraoperative difficulties in identifing a choledochal cyst. Retrospectively, an entire exploration, including elevation of the liver, should have been able to demonstrate a clear separation of the cystic structure from the liver. This intraoperative exploration should be performed and prompt any surgeon to dispute the preoperative diagnosis. Laparoscopic fenestration of a hepatic cyst is the appropriate approach. However, the finding of bile, and of even greater significance, two bile ducts, while possible, is so unusual for a hepatic cyst that it justifies an intraoperative re-evaluation by cholangiography. An intraoperative cholangiography in this case would have clarified the anatomy and pathology beyond any doubt.

## Conclusion

The case demonstrate the diagnostic and therapeutical difficulties in the treatment of choledochal cysts. To prevent such complications it is important to include the choledochal cyst firstly in the differential diagnosis.

## Competing interests

The author(s) declare that they have no competing interests.

## Authors' contributions

UW wrote the manuscript and did the literature search. DHB had a supervisory role. KB provided editorial assistance and was involved in the clinical treatment. All authors read and approved the final manuscript.

## Consent

The written informed consent of the patient was obtained for this publication.
